# Understanding patient views and experiences of the IDENTIfication of PALLiative care needs (IDENTI-PALL): a qualitative interview study

**DOI:** 10.3399/BJGP.2023.0071

**Published:** 2024-01-09

**Authors:** Isabel Leach, Catriona R Mayland, Nicola Turner, Sarah Mitchell

**Affiliations:** Division of Clinical Medicine, School of Medicine and Population Health, University of Sheffield, Sheffield.; Division of Clinical Medicine, School of Medicine and Population Health, University of Sheffield, Sheffield.; School of Health Sciences, University of Nottingham, Nottingham.; Division of Primary Care, Palliative Care and Public Health, University of Leeds, Leeds.

**Keywords:** end-of-life care, needs assessment, palliative care, patient perspectives, primary health care, qualitative research

## Abstract

**Background:**

Palliative care improves quality of life for people with life-threatening illnesses. There are longstanding inequalities in access to palliative care, with many people never identified as having palliative care needs, particularly frail older people, those with non-malignant disease, and people from ethnic minority backgrounds. Little is known about the process of identification of palliative care needs from a patient perspective.

**Aim:**

To provide new understanding into patient views and experiences of the process of identification of palliative care needs, and to explore the impact of identification on health care, if any, from a patient perspective.

**Design and setting:**

A qualitative interview study undertaken with patients and family carers in a major UK city.

**Method:**

Semi-structured interviews were carried out with patients (and/or family carers) identified as being on general practice palliative care registers. An inductive thematic analysis was conducted to explore the data.

**Results:**

Eleven participants were recruited: eight patients and three family carers. The following three interrelated themes were identified: 1) misconceptions about palliative care and unshared prognostic uncertainty hinder the identification of palliative care needs; 2) a compassionate, timely approach is required for identification of palliative care needs, with or without an identification tool; and 3) identification of palliative care needs is beneficial where it leads to proactive holistic care.

**Conclusion:**

A compassionate approach, sharing of prognostic uncertainty, and proactive primary care are key to timely, beneficial identification of palliative care needs. Future policy should ensure that identification is an adaptable, personalised process to meet the individual needs of people with advanced serious illnesses.

## Introduction

Palliative care improves the quality of life, symptom burden, and satisfaction with care for people with any life-threatening illness, particularly when introduced early in the disease trajectory.^1^^–^3 The number of people worldwide who could benefit from a palliative approach to their care is growing rapidly. As the population ages and health-related suffering increases, it is projected that 87% more people will need palliative care in the next 40 years.^4^

Early integration of palliative care is widely considered to be best practice.^5^ The World Health Organization advocates timely palliative care *‘to help patients live as actively as possible until death’* through the *‘correct assessment and treatment of pain and other problems, whether physical, psychosocial or spiritual’*.^6^ Yet many people, particularly frail older people, those with non-malignant disease, and people from ethnic minority backgrounds, do not receive palliative care at all before they die, or only receive it in the very final days of life.^7^^,^^8^

Primary care is key to universal palliative care.^9^^,^^10^ Early identification of palliative care needs has the potential to improve care, seeking out patients who could benefit from a comprehensive needs assessment focused on quality of life and what matters most to patients and families.^11^ However, the initiation of palliative care is nuanced and rarely straightforward for clinicians.^4^ Patients with non-malignant chronic illnesses and multimorbidity often follow an unpredictable illness trajectory with no clear palliative ‘phase’ of illness.^12^

There is growing interest in strategies to proactively search for patients with palliative care needs in the primary care setting.^13^^–^15 At least 10 different palliative care identification tools and processes have been developed for use in primary care, including the development of tools to search electronic patient records;^15^ however, there has been relatively little validation or evaluative research on these tools. To date, three systematic reviews have concluded that existing tools and processes are limited in their ability to accurately identify patients who could benefit from palliative care.^15^^–^17

Patient experiences should be central to the development and implementation of processes to identify palliative care needs, but research is limited. This study aimed to investigate the perspectives of patients with advanced, serious illnesses who had been identified as ‘palliative’, and any benefits or drawbacks they perceived of having their palliative care needs identified.

**Table:** How this fits in

There is a lack of understanding regarding patient views and experiences of identification of palliative care needs. This study aimed to provide a new understanding. It has suggested an individualised and compassionate approach is required, with key components including open conversations about palliative care and the sharing of prognostic uncertainty. Proactive palliative care intervention by primary healthcare professionals following identification of need is valued by patients and requires further attention in research, policy, and practice.

The aims were as follows:

1. to understand the experience of patients in the process of identification of their palliative care needs and how this could be improved, including using a tool to support the process; and
2. to understand how identification of palliative care needs impacts on the current and future health care of patients, positively or negatively.

## Method

### Study design

Patient experience of palliative care is nuanced and highly individual. This study was designed to understand this experience and therefore required a methodological approach commensurate with this complexity. Qualitative methods were considered the most appropriate to understand individual patient experience, with data collection through semi-structured interviews with patients and their family carers.^18^ Given the potentially sensitive subject area and new insights emerging throughout data collection, an ongoing process of reflexivity was required. The methods allowed for rapport building, flexibility, and the time and space to gather rich insights into participants’ views and experiences. The research protocol has been published elsewhere.^19^

### Study setting

This was a primary care-based study engaging with general practices across Sheffield, South Yorkshire, in the UK. Sheffield is a diverse city with pockets of both extreme affluence and areas of significant socioeconomic deprivation. At the time of the study, there were 80 general practices in Sheffield arranged into 15 primary care networks.

### Population

All patient participants were registered with a general practice, had previously been identified as ‘palliative’ by their primary care team, and were included on individual practices’ palliative care registers. Family carers participated if they were invited to do so by the patient participant. Inclusion and exclusion criteria are outlined in Box 1.

**Box 1.** Inclusion and exclusion criteria for interviews

Inclusion criteria	Adults (aged ≥18 years) with advanced serious illness under the care of a GP who either: receive input from specialist palliative care services; and/or are aware of (that is, had discussions about) palliative care, including their inclusion on the individual practice palliative care register. Family carers (aged ≥18 years) of an eligible adult who has agreed to take part in an interview.
Exclusion criteria	Children and young people aged <18 years. Adults with advanced serious illness who are unable to participate in a conversational interview for any reason related to their condition. Adults who are unable to provide informed consent in English. Family carers who have not been invited to take part in an interview by the patient participant.

### Recruitment and sampling

Participants were recruited from a range of geographical areas across Sheffield with contrasting demographics to maximise the diversity of participant experiences. Recruitment was via clinical teams. Doctors and community nurses were asked to identify up to two eligible patients and introduce the study to them during a routine consultation, or by telephone. Verbal consent was obtained from interested patients for their contact details to be shared with the research team.

The main researcher (lead author) contacted interested participants directly to introduce herself and the project, share participant information resources, and organise an interview. Informed consent was either obtained virtually via an electronic form or in person with a paper form, depending on participant preference. All participants were given the opportunity to discuss the study and ask any questions before providing consent.

### Data collection

Interviews were conducted between January and May 2022 by the lead author, a female medical student with experience and training in qualitative research methods. According to participant preference, interviews took place face-to-face at the patient’s home or over the telephone, and were conducted either with the patient individually or alongside their family carer. Interviews were audio-recorded, and field notes were made to capture observations and reflections.

Demographic data (age, sex, ethnic group, and postcode) were collected at the beginning of each interview to provide contextual information about each participant. The 2019 Index of Multiple Deprivation (IMD) was used to calculate the level of socioeconomic deprivation for each postcode.^20^

The interview topic guide (Box 2) was developed following a literature review and using insights gained from patient and public involvement (PPI) work conducted with patient and carer representatives from the University of Sheffield Palliative Care Studies Advisory Group. The guide was designed to encourage participants to develop their own account. The coherence and phrasing of questions were tested in the first interview where two authors were both present; questions were then adapted accordingly. Passive interviewing, including the use of open questions, allowed the participants time to reflect on their experiences. Alongside the topic guide, interview techniques were employed including summarising and reflecting, active listening, and use of silence.

**Box 2.** Topic guide for interviews

**Question**	**Prompts**
Can you tell me a little about yourself and your story?	
What does palliative care mean to you?	The World Health Organization (WHO) states that: *‘Palliative care improves the quality of life of patients and that of their families who are facing challenges associated with life-threatening illness, whether physical, psychological, social or spiritual. The quality of life of caregivers improves as well.’*^6^ What do you think about this definition? Do you agree or disagree with it?
Do you remember when you first heard the term ‘palliative care’?	Do you remember a particular conversation when palliative care was first discussed with you? Who discussed this with you? What triggered the discussion? Was it a particular event? Was it about a referral to a specialist palliative care service? Had you thought of ‘palliative care’ before a professional talked to you about it?
How did you feel when you were identified as having palliative care needs?	Positive or negative feelings? Have you experienced any negative effects or had any negative experiences?
Are there any benefits you can describe about having your ‘palliative care’ needs identified?	Personal benefits? Benefits to your care?
Do you know whether you are on a general practice palliative care register?	If yes: Did your GP ask whether you could be included on the register? Are there any benefits to being on the register? If no: Do you think there would be any benefit to being on a register? What benefits would you consider to be most important?
There is a lot of work happening to develop tools to identify patients who have palliative care needs through a computer search. How would you feel if your GP identified that you had palliative care needs this way?	Positive or negative feelings? What advice would you give to GPs or those developing these tools about how it feels as a patient to be identified?
How would you want your GP to communicate with you that you had been identified as having palliative care needs?	Would you prefer to find out via a letter, text, phone call, or a face-to-face appointment? What information would you want to be told about palliative care?
Have you any other thoughts or reflections you would like to share?	Thank you for sharing your experiences.

### Data management

Interview recordings and field notes were transcribed verbatim by the lead author. Transcripts were anonymised and uploaded into NVivo. Transcripts were not returned to participants for validation to minimise the potential burden in taking part in the study, particularly for participants with limited life expectancy and varied levels of literacy.

### Data analysis

Coding of the data began alongside data collection, with early familiarisation with the data, reflection, and note taking. An inductive and iterative approach was taken, as described by Braun and Clarke.^21^ Codes were assigned to every item of data, then grouped into broad overarching themes.^22^ Preliminary concepts were discussed at regular meetings with the research team in order to develop the themes and to decrease lone researcher bias.^21^ Two authors independently coded a selection of transcripts, allowing for comparison and further development of codes and themes.

## Results

### Study population

In total, 11 participants were recruited: eight patients and three family carers. Patient participants had a variety of medical conditions (categorised as malignant or non-malignant for anonymity) and ranged in age from 51–87 years (median of 74 years). All participants were White British. Nine of the eleven participants lived in an area classified within the top two deciles of deprivation according to the 2019 IMD.^20^

There were eight further patient participants who expressed an interest in taking part in the study and consented for their contact details to be passed onto the research team. However, they each became too unwell to participate, or died before an interview could be arranged.

Interviews ranged in duration from 28–64 minutes, with a median time of 43 minutes. Participant characteristics are detailed in Table 1.

**Table 1.** Patient demographics and interview details

**Interview number**	**Participants present at interview**	**Participant identifier**	**Age at recruitment, years**	**Sex**	**Primary medical condition(s)**	**Interview location**	**IMD decile^a^**
1	Patient	P001	77	M	Non-malignant	Patient’s home	1
2	Patient	P002	73	F	Non-malignant	Care home	2
3	Patient	P003	51	F	Malignant	Over the telephone	4
4	Patient	P004	80	M	Non-malignant	Patient’s home	2
	Family carer	C004	74	F	N/A		
5	Patient	P005	57	F	Malignant	Over the telephone	6
6	Patient	P006	65	M	Malignant	Patient’s home	2
	Family carer	C006	58	F	N/A		
7	Patient	P007	87	M	Non-malignant	Patient’s home	1
	Family carer	C007	76	F	N/A		
8	Patient	P008	75	M	Malignant and non-malignant	Patient’s home	1

a *1 indicates highest decile of deprivation. F = female. IMD = Index of Multiple Deprivation. M = male. N/A = not available.*

### Qualitative findings

Experiences of the identification of palliative care needs varied hugely between participants, reflecting the individuality of patients’ stories, and their diverse perspectives and understanding of their conditions and the care they had received. The following three interconnected themes, related to identification of need, were identified: 1) misconceptions about palliative care and unshared prognostic uncertainty hinder the identification of palliative care needs; 2) a compassionate, timely approach is required for identification of palliative care needs, with or without an identification tool; and 3) identification of palliative care needs is beneficial where it leads to proactive holistic care.

### Theme 1: Misconceptions about palliative care and unshared prognostic uncertainty hinder the identification of palliative care needs

Understanding of the terminology of palliative care, including perceptions of who it is for and when it is delivered, underpinned experiences. There was a sense that people were *‘frightened’* (P005, patient, female [F], aged 57 years, IMD decile 6) of being ‘labelled’ as someone with palliative care needs owing to its connotations with death. Almost all participants associated palliative care with care at the very end of life, often in a hospice, when one is on their *‘last legs’* (P006, patient, male [M], aged 65 years, IMD decile 2). Participants with cancer tended to associate palliative care with a distinct service:

*‘The oncologist gives you the treatment, and the palliative team get you through it.’*

(P003, patient, F, aged 51 years, IMD decile 4)

For several participants, use of the term ‘palliative care’ during the interview evoked concerned or confused responses. Some had no concept of palliative care at all, despite the specific inclusion criteria for the study. Sometimes phrasing questions using ‘quality-of-life care’ or ‘supportive care’ was better received. Participants reported a tendency to avoid directly talking about palliative care because *‘it’s got a stigma’* (C006, family carer, F, aged 58 years, IMD decile 2).

Participants who had not been referred to a specialist palliative care team and received palliative support from primary care alone struggled to articulate their experiences of identification; many were adamant they did not receive palliative care at all. Participant understanding of their medical condition(s) and prognosis greatly affected how they recounted their experiences of identification of palliative care need. Many felt frustrated by a lack of honest and open communication with healthcare professionals about their condition:

*‘The doctors didn’t explain it to me … I didn’t realise how ill I’d been. Everything … my kidneys and my liver and my heart … they were all on the verge of giving up. But the doctors didn’t explain it to me that well … They just came round every day and said, “We’ll see you tomorrow, we’ll see you tomorrow.”’*

(P002, patient, F, aged 73 years, IMD decile 2)

Participants emphasised the importance of sharing uncertainty about prognosis before discussions about palliative care were held. Multiple participants felt unheard when they raised concerns about the progression of chronic disease, being told everything was *‘under control’* (P001, patient, M aged 77 years, IMD decile 1) instead. The consequences of such communication surrounding prognosis was highlighted by a family carer who *‘didn’t really grasp what were the matter with* [her husband]*’* until she eventually *‘got the impression that* [he was] *at the end of his life’* (C007, family carer, F, aged 76 years, IMD decile 1):

*‘I think the day* [the GP] *came and said stop all his medication, I would have liked her to explain to me why, you know. But she didn’t. She just said stop all his meds, just stop it, you know.’*

(C007, family carer, F, aged 76 years, IMD decile 1)

P003 (patient, F, aged 51 years, IMD decile 4) vividly described feeling *‘shocked’* when a member of the palliative care team first contacted them because they did not understand, or perhaps had not yet processed, their prognosis:

*‘I’m being totally honest with you now, part of the reason that it shocked me when* [name of palliative care nurse] *rang, was because I didn’t fully understand my diagnosis. I knew that I’d got secondary* [organ] *cancer, but I didn’t understand that it actually meant it will kill you.’*

(P003, patient, F, aged 51 years, IMD decile 4)

### Theme 2: A compassionate, timely approach is required for identification of palliative care needs, with or without an identification tool

For many participants, palliative care was explicitly discussed for the first time by their GP alongside referral to a specialist service. The stage of illness in which these conversations took place varied. One participant, who was unaware of their own palliative care needs, was referred to a specialist team following a prolonged period of poor symptom control where the GP had *‘prescribed every possible painkiller that* [P004, patient, M, aged 80 years, IMD decile 2] *could have’* (C004, family carer, F, aged 74 years, IMD decile 2).

In contrast, timely referral to specialist services meant one participant was able to receive care at home from the community hospice team for >18 months, which was a huge source of support for them and their family:

*‘It were a locum GP. He were very very nice that gentleman. And he said, “I think we better get some more people involved”, you know because he is going down … and one of them were* [name of hospice] *what he mentioned.’*

(C007, family carer, F, aged 76 years, IMD decile 1)

Participants felt that a more formal process for identification of need could have a positive impact on their care, and were open to the idea that an identification tool could be used to guide the process:

*‘Well, first of all I’d have a shock them people* [at the GP surgery] *telling me they are doing something* [to try to identify patients with palliative care needs]*.* [But then] *I wouldn’t mind at all.’*

(P001, patient, M, aged 77 years, IMD decile 1)

While the potential benefits of a tool to guide the identification process were considered, participants also emphasised that automation of such a nuanced area of practice *‘might miss people’* (P003, patient, F, aged 51 years, IMD decile 4) and that the convenience of a tool should not compromise the quality of care:

*‘At first* [a palliative care identification tool] *doesn’t sound very person centred or anything, does it? But there has to be ways doesn’t there? It’s probably a good thing because how would they do it otherwise? Somebody trawling through the records, you know, one by one … well that’s not time or cost-effective for anybody is it?’*

(P005, patient, F, aged 57 years, IMD decile 6)

Regardless of conflicting opinions on identification tools, all participants highlighted the importance of compassionate communication. This was lacking for many:

*‘The only thing I found weird was how I initially found out about* [needing palliative care]*. But that could have just been a mixture of me not quite understanding the diagnosis, and all the information being given to me in leaflets and a book … I definitely want somebody to talk to me about things like that, rather than finding out through a leaflet … because you can’t ask a leaflet questions!’*

(P003, patient, F, aged 51 years, IMD decile 4)

The need for time and space to process information, with opportunity to ask questions, was specifically described as important:

*‘So, if somebody actually sat you down and told you face to face, who, what, when, where, and how. I think that would be better received and better understood. Because if anybody had the same response as me, they’d be able to go “why?” and they’d be able to explain it properly.’*

(P003, patient, F, aged 51 years, IMD decile 4)

### Theme 3: Identification of palliative care needs is beneficial where it leads to proactive holistic care

Implications of identification of palliative care needs for healthcare experiences varied between participants. Participants who had been referred to specialist palliative care services described the support they had received as a positive and beneficial aspect of their care. The most described benefit of identification was a sense that *‘someone* [was always] *there*’ (P003, patient, F, aged 51 years, IMD decile 4) to offer both physical and emotional support:

*‘I know if I have any questions … all I need to do is make a phone call and somebody* [at the hospice] *will tell me what’s going on, or they’ll help me sus out why something is happening. So, it’s good to know that I’ve got that backup there now.’*

(P003, patient, F, aged 51 years, IMD decile 4)

Identification of palliative care needs often acted as a catalyst for more holistic care, particularly for patients with cancer. Participants recognised a shift away from biomedical, symptom-oriented care coinciding with referral to specialist palliative care teams:

*‘On the next appointment, the first thing* [name of palliative care consultant] *said was, “How was Christmas? Because I know you were anxious about it.” And I was like oh God, she is talking to me … she’s not just talking to all these symptoms.’*

(P005, patient, F, aged 57 years, IMD decile 6)

P003 (patient, F, aged 51 years, IMD decile 4) and P005 (patient, F, aged 57 years, IMD decile 6), who both had cancer, talked exclusively about the benefits of palliative care in the context of specialist services. When asked specifically about the involvement of their primary care teams in their palliative care, they both described not having any need for them. However, they did recognise that they were able to access support from general practice much more quickly following identification, which is a key benefit of inclusion on the practice’s palliative care register.

Other participants, particularly those with non-malignant disease and multimorbidity, described their GP as an integral part of their palliative care:

*‘*[Name of GP] *rings up every fortnight. Always asking how* [P006, patient, M, aged 65 years] *is, does he need any pain relief? Am I OK? Do I need anything? … He always rings up, yeah.’*

(C006, family carer, F, aged 58 years, IMD decile 2)

Proactive primary care and trusted relationships offered participants and their families a sense of security at times of great vulnerability. This was particularly crucial for participants who did not receive care from specialist palliative care teams.

Notably, one participant did not believe that the high quality of care they had received from their primary care team was a consequence of their palliative ‘label’, but rather the *‘good relationship’* (P008, patient, M, aged 75 years, IMD decile 1) they had built with their primary care team over many years:

*‘I did look* [at my advanced care plan] *the other week and I thought this seems a bit meaningless this. You know what I mean? It’s like dutiful things that people seem to have to do for some reason and they’re there. So, it’s everybody must have a care plan … everybody blah blah. No seriously, and people are different like. I keep getting said to me, people are different. Well, if they are different, well treat them bloody differently then!’*

(P008, patient, M, aged 75 years, IMD decile 1)

While numerous positive experiences were described, some participants received minimal support from their primary care team, nor any specialist palliative care service, following identification of their palliative care needs. This caused a sense of feeling *‘not supported at all’* (C004, family carer, F, aged 74 years, IMD decile 2).

One participant did not believe they received any palliative support, saying they must be *‘at the bottom’* of their GP practice’s palliative care register. They explained that they would benefit from regular check-ins from their GP, echoing the benefits of proactive primary care described by other participants:

*‘It would be nice if somebody would say, “Well I’m just phoning up to see whether you’re still living or not?” I’d say, “Well I’m sorry to tell you that I am.” No, it would be nice, just to get a phone call! Do you know what I mean?’*

(P001, patient, M, aged 77 years, IMD decile 1)

## Discussion

### Summary

This study has provided new insights into the experiences of adults with advanced serious illness and their family carers in the identification of their palliative care needs, and the impact this had on their health care.

Many participants, particularly those with non-malignant disease, did not relate to having palliative care needs even though this was a specific consideration in the inclusion criteria for participation in the study. Reasons for this lack of awareness included misconceptions about palliative care and unshared prognostic uncertainty between healthcare professionals and patients, alongside stigma surrounding conversations about death and dying. Participants who were able to recount their experience of identification often described it purely in the context of referral to specialist services, even when, on further questioning, their primary care team had previously been a significant source of palliative support in the community.

Proactive primary care and an individualised, compassionate approach were key to the process of identification of palliative care needs from a patient perspective. Time for meaningful and open conversations was recognised as a priority regardless of the route to identification of need.

### Strengths and limitations

A methodological strength of the study was the qualitative approach, allowing in-depth data to be gathered. Rapport with the researcher encouraged participants to provide detailed and candid accounts with both positive and negative aspects of care shared freely. As expected, each participant’s story was highly individual owing to the diversity in medical conditions and variety in type and quality of care they had received. It is likely that new insights and experiences would have been recorded if the interviews had continued. Nevertheless, data saturation was achieved around the main themes, particularly surrounding the benefits of proactive holistic care following identification and the experiences of communicating with healthcare professionals about prognoses.

There appeared to be value in a student conducting interviews rather than a healthcare professional actively engaged in palliative care. Participants were made aware on introduction of the study that the lead author was undertaking the interviews as part of her intercalated degree; all participants were pleased to contribute to the project, which acted as a strong foundation for building rapport. Regular discussion with the research team was important to support the lead author in the process and decreased lone researcher bias in the analysis.

Recruitment to this study was challenging owing to the unpredictable, palliative nature of the conditions of participants, and depended on the interest of clinical teams. Recruitment from primary care meant that the study population was not limited to participants who received specialist palliative care. This allowed for exploration of the complexity of the process of identification of palliative care needs within primary care.

There was diversity in the study population in terms of patients’ medical conditions and experiences of care. However, all participants were White British, aged ≥50 years, and, within the resource constraints of this study, only patients who could speak English were eligible to participate. Research to explore the experiences of non-English speakers, adults aged 18–50 years, and people from ethnically diverse communities is vital. Further research focusing on the complexity of identification in frail older people, including the views and experiences of patients in this cohort, is also necessary given the ageing population.

### Comparison with existing literature

There is limited patient-focused research in this subject area. This study has contributed to a small body of existing research seeking to understand patient views and experience of identification. Most of the current evidence base focuses on the development of identification tools rather than the complexity of the identification process and the potential impact on a patient’s health care.

Participants in this study were generally receptive to the need to enhance the process of identification of palliative care needs, including using palliative care identification tools in general practice, providing that their use would lead to meaningful conversations about patient priorities and preferences for care. These findings are consistent with previous research into patient views of identification tools, which reported their use could be beneficial when used with empathy and alongside the personal and clinical judgement of healthcare professionals.^23^^–^25

GPs have previously described the importance of time when caring for patients with palliative care needs.^26^ Participants in the present study agreed that time and frequent check-ins with a member of their primary care team gave patients and families the opportunity to ask questions and feel supported, even when there was no immediate medical concern. Previous studies have also shown that continuity of care with the same GP is associated with reduced hospital admissions at the end of life, further demonstrating the potential impact of proactive palliative care interventions by primary healthcare professionals following identification of need.^27^^,^^28^

Although many participants highly valued and relied on their close relationship with their GP, few directly recognised this as a source of ‘palliative’ support. For most, only a formal referral to specialist palliative care teams constituted identification of need. This supports findings from previous research that demonstrated the roles of primary healthcare professionals were unclear to patients in the delivery of palliative care, and is highly relevant to future policy and service design in delivering effective primary palliative care.^29^

### Implications for research and practice

Honest and compassionate discussions surrounding prognosis and any uncertainty are necessary to initiate palliative care earlier in the disease trajectory alongside other medical treatments. The sensitivity and complexity of these conversations are key factors to consider as tools to enhance the identification process are developed and implemented into general practice.

For many patients, particularly those with non-malignant multimorbidity and frailty, the ‘right’ time to introduce palliative care is unclear.^12^^,^^30^ Identification tools may be helpful, but more research is required to understand effective implementation of such tools, and the impact on future care for patients.

Participants in this study valued the input of primary care and emphasised the importance of time for compassionate communication at the point of identification of palliative care needs. This finding requires more focus in current primary care environments where conflicting demands mean that time for conversations about palliative care are limited.^26^ A sense of security can be provided through proactive primary care, and this should be considered as new models of primary care are developed.
